# Broncholithiasis: Diagnostic and Therapeutic Insights From a Contemporary Case Series of Five Patients

**DOI:** 10.7759/cureus.106167

**Published:** 2026-03-30

**Authors:** Wilson S Peñafiel-Pallares, Jorge Valdivieso-Tenemaza, Juan S Salamanca-Espitia, Matthew McCoy, Diego Navarrete-Navarrete, Ojas Thapa, Ashish Maskey

**Affiliations:** 1 General Practice, Universidad de las Américas, Quito, ECU; 2 Internal Medicine - Pediatrics Program, Marshall University Joan C. Edwards School of Medicine, Huntington, USA; 3 Pulmonary, Critical Care and Sleep Medicine, University of Kentucky College of Medicine, Lexington, USA; 4 General Surgery, UMass Chan Medical School, Worcester, USA; 5 Pulmonary and Critical Care, University of Kentucky, Lexington, USA

**Keywords:** broncholithiasis, hemoptysis of endobronchial and parenchymatous origin, interventional bronchoscopy, pulmonary calcification, tracheobronchial tree

## Abstract

Broncholithiasis refers to the presence of calcified or ossified material within the tracheobronchial tree. This rare condition is more frequently reported in regions endemic for tuberculosis and histoplasmosis, where necrotizing granulomatous lymphadenitis may lead to dystrophic calcification and eventual erosion of calcified lymph nodes into the bronchial lumen. Clinical presentation is heterogeneous and ranges from incidental findings to hemoptysis, chronic cough, recurrent infection, and post-obstructive complications. We conducted a retrospective review of five patients diagnosed with broncholithiasis at our tertiary care center. Clinical records were reviewed for demographic characteristics, presenting symptoms, imaging findings, bronchoscopic interventions, microbiologic data when available, and short-term follow-up outcomes. Two patients presented primarily with hemoptysis, one with chronic cough and severe infectious complications, one with recurrent pneumonia and lobar collapse, and one was incidentally referred with an obstructing broncholith. All five cases were managed bronchoscopically using combinations of rigid or flexible bronchoscopy, forceps, balloon dilation, cryotherapy, topical hemostatic measures, and argon plasma coagulation when needed. Two patients also required video-assisted thoracoscopic surgery for empyema management rather than for broncholith extraction itself. Where available, microbiologic findings included *Actinomyces* and mixed molds, highlighting the risk of post-obstructive infection. These cases underscore the heterogeneous clinical spectrum of broncholithiasis and support bronchoscopy as the primary diagnostic and therapeutic modality in appropriately selected patients. Early recognition, multidisciplinary management, and attention to associated infectious complications may improve outcomes.

## Introduction

Broncholithiasis is a rare condition defined by the presence of calcified or ossified material in the tracheobronchial tree. It is most prevalent in regions endemic to tuberculosis and histoplasmosis, which are the leading causes of the condition [1]. These infections result in necrotizing granulomatous lymphadenitis and subsequent dystrophic calcification. This process may lead to the erosion of calcified lymph nodes into the bronchial lumen or the in situ calcification of foreign bodies [1,2].

Although frequently asymptomatic, broncholithiasis can manifest as chronic cough, hemoptysis, or recurrent infections. Early diagnosis and appropriate management are essential to prevent morbidity [1-3]. This study presents five cases of broncholithiasis diagnosed and treated at our institution, focusing on their clinical presentations, bronchoscopic management, and outcomes.

We performed a retrospective review of five patients diagnosed with broncholithiasis at our tertiary care center. Clinical records were reviewed for demographic data, presenting symptoms, imaging findings, presumed or confirmed etiology when available, microbiologic or pathologic results, bronchoscopic technique, adjunctive interventions, and follow-up outcomes. All patients underwent diagnostic and/or therapeutic bronchoscopy. The type of bronchoscope used (rigid or flexible), adjunctive tools (forceps, balloon dilation, cryotherapy, argon plasma coagulation, and topical hemostatic agents), and peri-procedural outcomes were recorded. Available follow-up consisted of outpatient clinical assessment and interval imaging when documented in the medical record.

## Case presentation

Case 1

**Figure 1:**
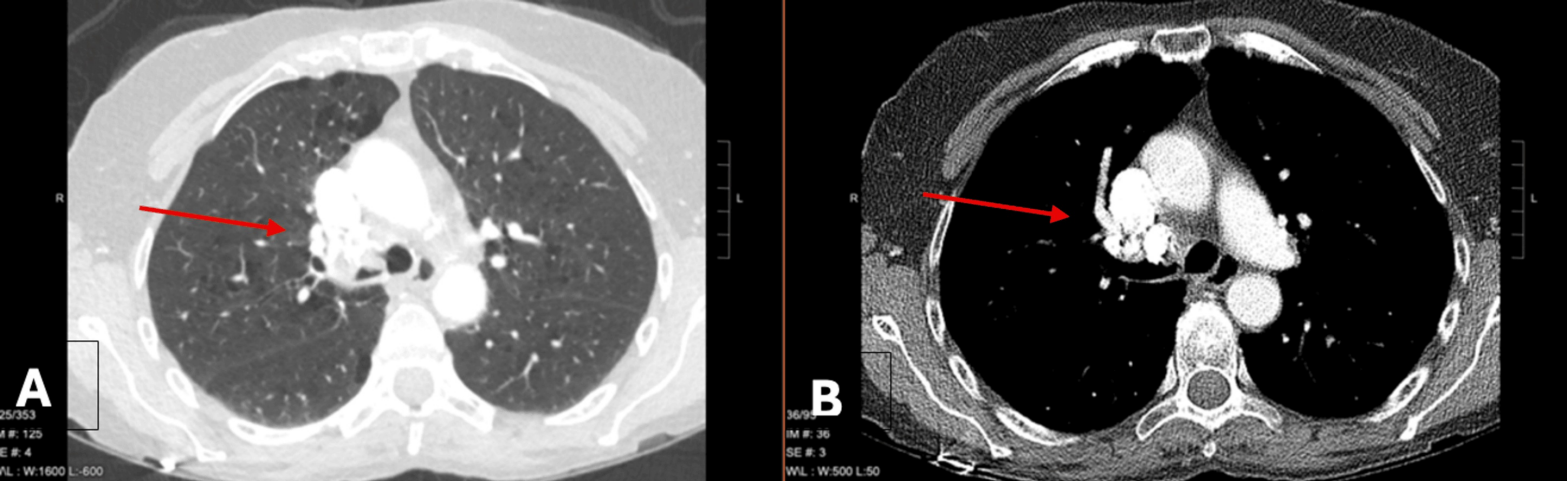
Computed tomography findings of broncholithiasis in Case 1. Axial contrast-enhanced chest CT images displayed in lung window (A) and mediastinal window (B) demonstrate calcified mediastinal lymphadenopathy with a large, densely calcified station 4R lymph node protruding into the right mainstem bronchus, resulting in significant airway narrowing (arrows). These findings are consistent with broncholithiasis secondary to prior granulomatous disease.

Ms. B, a 70-year-old female with chronic obstructive pulmonary disease, rheumatoid arthritis, and a remote history of histoplasmosis, presented with two years of hemoptysis. Imaging revealed calcified mediastinal lymphadenopathy and a large, calcified station 4R lymph node protruding into the right mainstem bronchus (RMSB) (Figure 1).

**Figure 2:**
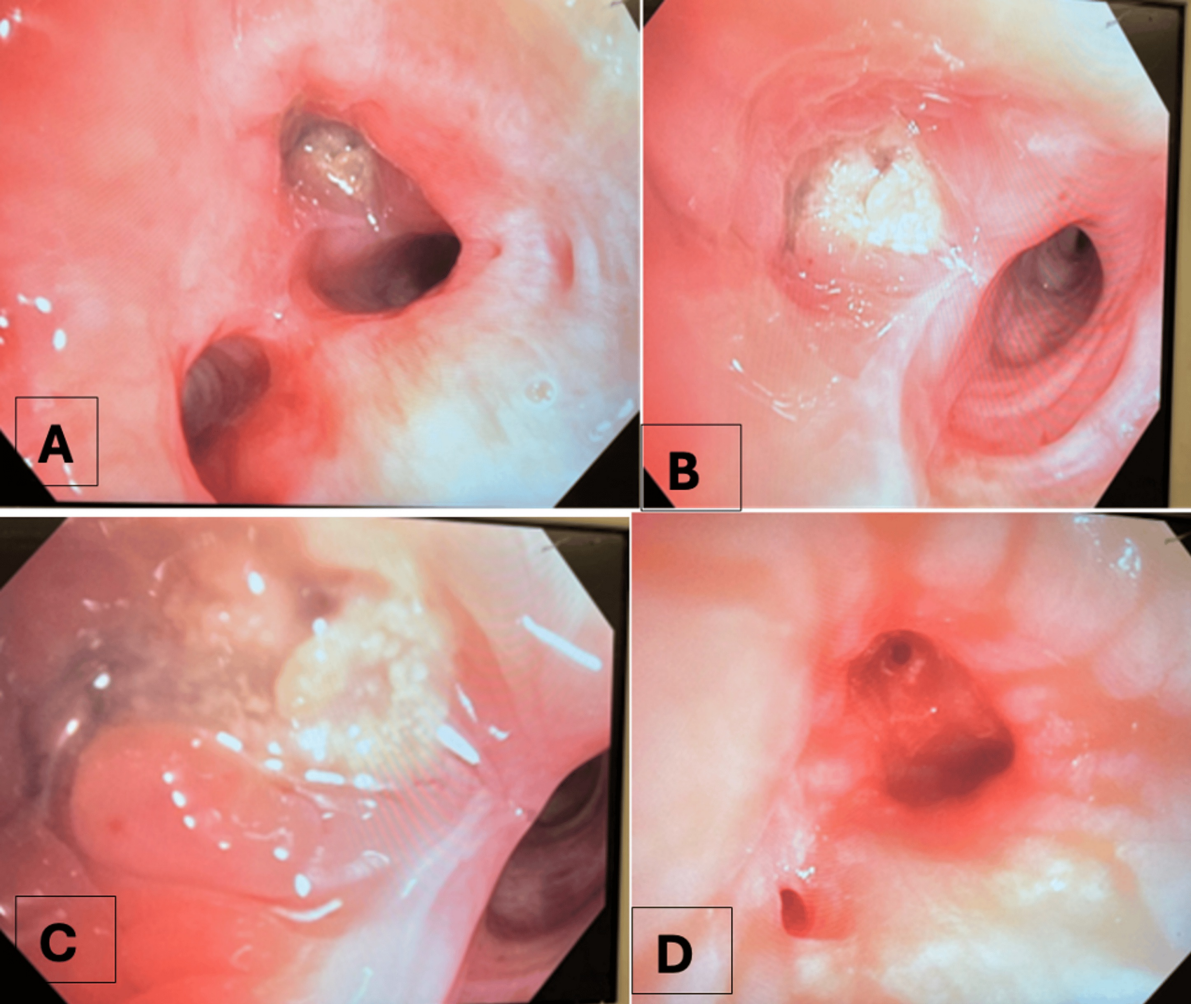
Bronchoscopic findings and stepwise management of broncholithiasis in Case 1. (A) Endoscopic view demonstrating a calcified broncholith protruding into the right mainstem bronchus (RMSB), causing significant luminal obstruction with surrounding granulation tissue. (B) After cryoablation (freeze–thaw technique), partial exposure of the broncholith with improved visualization of the obstructed airway. (C) Piecemeal extraction of the broncholith using rigid rat-tooth forceps, revealing the underlying bronchial wall. (D) Post-extraction view showing restoration of airway patency with mild mucosal erythema and a residual sinus tract following complete broncholith removal.

She underwent rigid bronchoscopy with an 11 mm Dumon scope. The granulation tissue obstructing 80% of the RMSB was treated with cryoablation (freeze-thaw method), followed by piecemeal removal of the broncholith using rigid rat-tooth forceps (Figure 2). Hemostasis was achieved with diluted epinephrine (1:10,000) and argon plasma coagulation (APC) (0.5 L/min, 20 W). Additional calcified fragments were removed with Blue Boston Scientific forceps (Boston Scientific, Marlborough, MA).

After complete removal, a 5 mm sinus tract was visible in the distal trachea (Figure 2D). Ms. B experienced resolution of hemoptysis and improvement in dyspnea at two-week and six-month follow-ups.

Case 2

**Figure 3:**
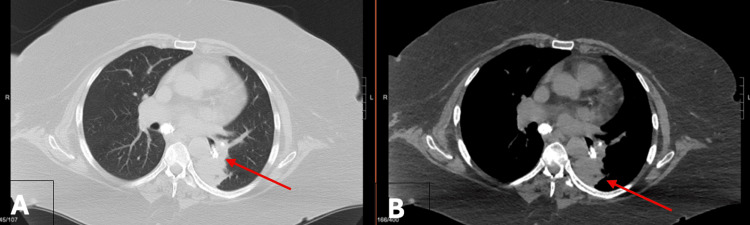
Computed tomography findings of broncholithiasis in Case 2. Axial contrast-enhanced chest CT images displayed in lung window (A) and mediastinal window (B) demonstrate calcified mediastinal lymphadenopathy with broncholiths obstructing the left lower lobe bronchus (arrows). Associated post-obstructive changes are evident, correlating with the patient’s clinical presentation of hemoptysis and recurrent infection.

Mrs. C, a 60-year-old female with cirrhosis, asthma, obstructive sleep apnea, and diabetes, presented with hemoptysis and a new left lower lobe (LLL) mass. Computed tomography demonstrated broncholiths obstructing the LLL with calcified mediastinal lymphadenopathy (Figure 3).

**Figure 4:**
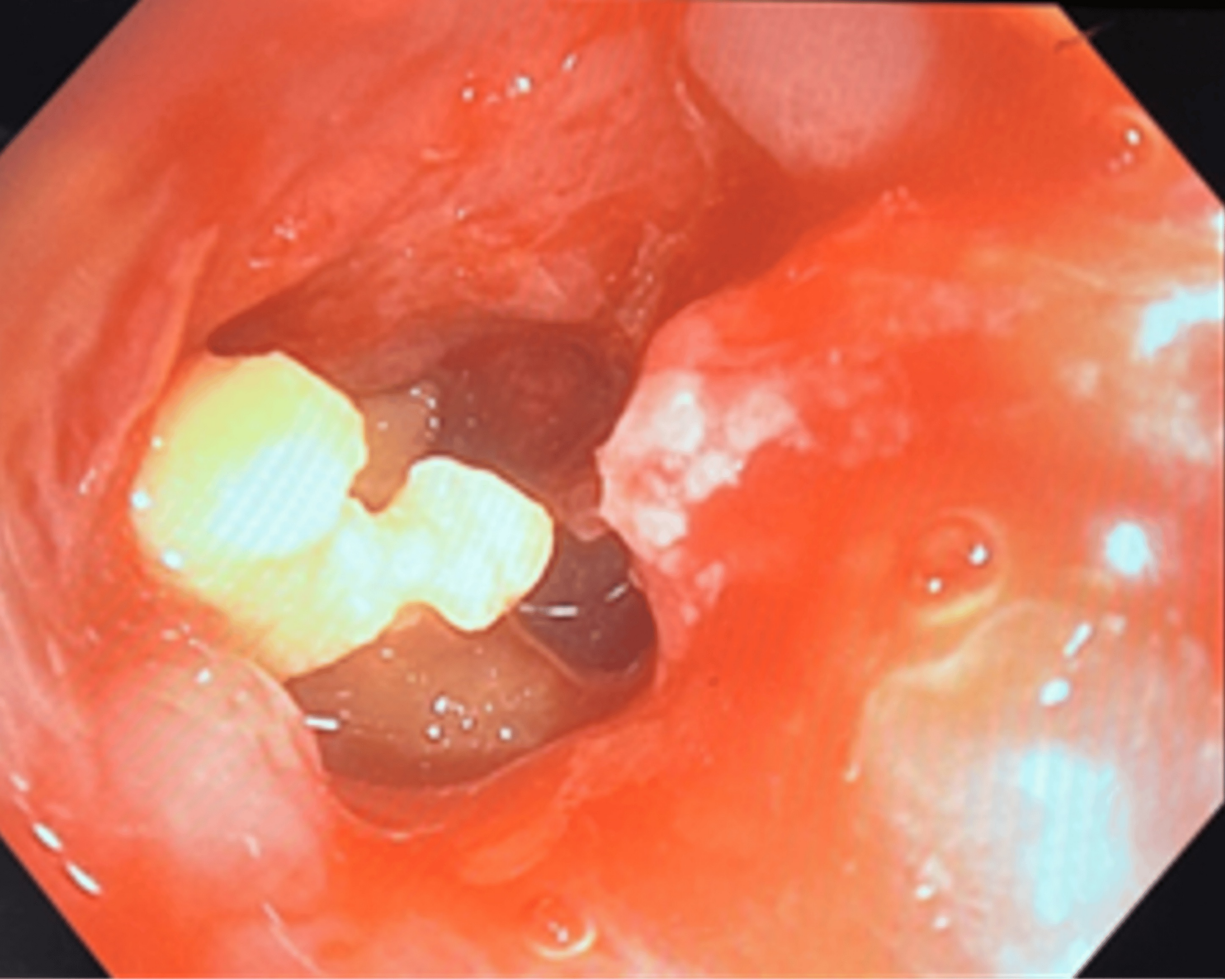
Bronchoscopic findings in Case 2. Flexible bronchoscopy demonstrating a densely calcified broncholith causing complete (100%) obstruction of the superior segment of the left lower lobe (LLL). The surrounding bronchial mucosa appears erythematous and friable, consistent with chronic inflammation and bronchomalacia identified after balloon dilation.

Initial flexible bronchoscopy revealed 100% obstruction of the LLL superior segment (Figure 4). Balloon dilation with a controlled radial expansion (CRE) balloon (8-9-10 mm) was performed, revealing significant bronchomalacia and an underlying broncholith (Figure 4). Cultures grew *Actinomyces* and mixed molds, prompting ampicillin-sulbactam therapy followed by amoxicillin-clavulanate.

Five months later, she was re-admitted for recurrent hemoptysis and COVID-19 infection. Repeat bronchoscopy showed friable mucosa and persistent obstruction. Another balloon dilation to 8 mm was performed with temporary visualization of the broncholith; however, due to poor visibility, extraction was aborted.

The patient developed a loculated pleural effusion, requiring chest tube drainage and video-assisted thoracoscopic surgery (VATS) decortication for empyema. Mrs. C completed a six-month doxycycline course for *Actinomyces* and demonstrated gradual symptom improvement.

Case 3

**Figure 5:**
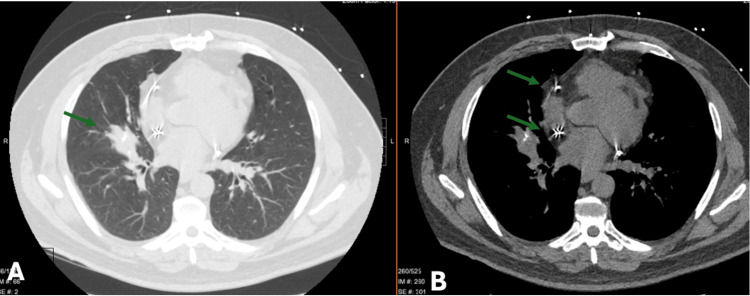
Computed tomography findings of broncholithiasis in Case 3. Axial contrast-enhanced chest CT images displayed in lung window (A) and mediastinal window (B) demonstrate a calcified broncholith obstructing the right middle lobe (RML) bronchus (green arrows). Associated focal airway narrowing is evident, correlating with the patient’s obstructive symptoms.

Mr. D, a 61-year-old male with coronary artery disease and prior pancreatitis complicated by a pseudocyst, was referred for an obstructing broncholith in the right middle lobe (RML) (Figure 5).

**Figure 6:**
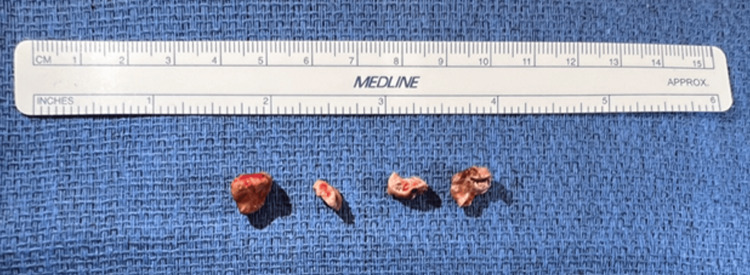
Extracted broncholith fragments in Case 3. Gross specimen photograph showing multiple calcified broncholith fragments removed piecemeal during rigid bronchoscopy. A measurement ruler is included for size reference, illustrating the irregular morphology and variable fragment dimensions following partial broncholith extraction.

During rigid bronchoscopy, manipulation caused bleeding from the RML, controlled with cold saline lavage and diluted epinephrine. The broncholith was partially removed in fragments (Figure 6) after airway dilation with a 5 Fr Fogarty balloon using 1.8 mm and 2.2 mm forceps. Hemostasis was achieved with thrombin (5000 U), epinephrine, and cold saline lavage.

Case 4

**Figure 7:**
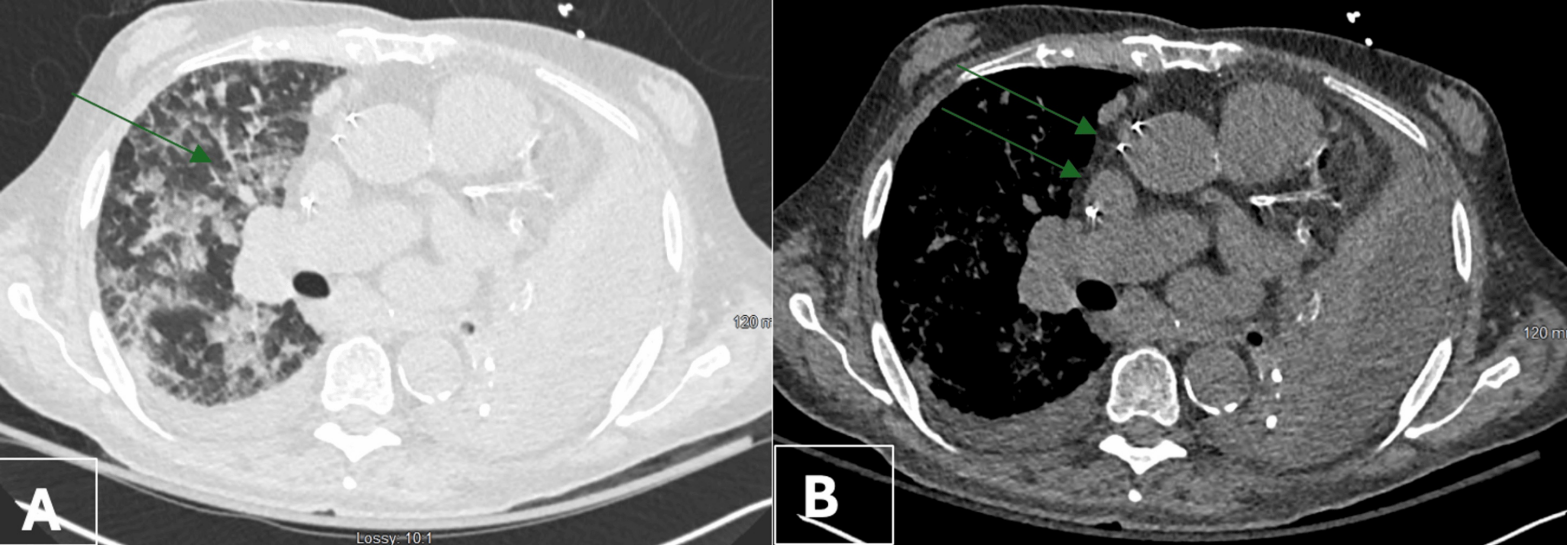
Computed tomography findings in Case 4. Axial contrast-enhanced chest CT images demonstrate left lower lobe collapse prior to intervention, consistent with post-obstructive pneumonia (A, lung window, arrow). Post-intervention imaging (B, mediastinal window) shows restoration of airway patency and re-expansion of the left lower lobe following bronchoscopic removal of an obstructing broncholith (arrows).

Mr. G, an 82-year-old male, presented with recurrent pneumonia and LLL collapse. Pre-procedurally, a left thoracentesis was performed. Flexible bronchoscopy revealed occluded LLL proper and superior segment with a large adherent broncholith (Figure 7).

**Figure 8:**
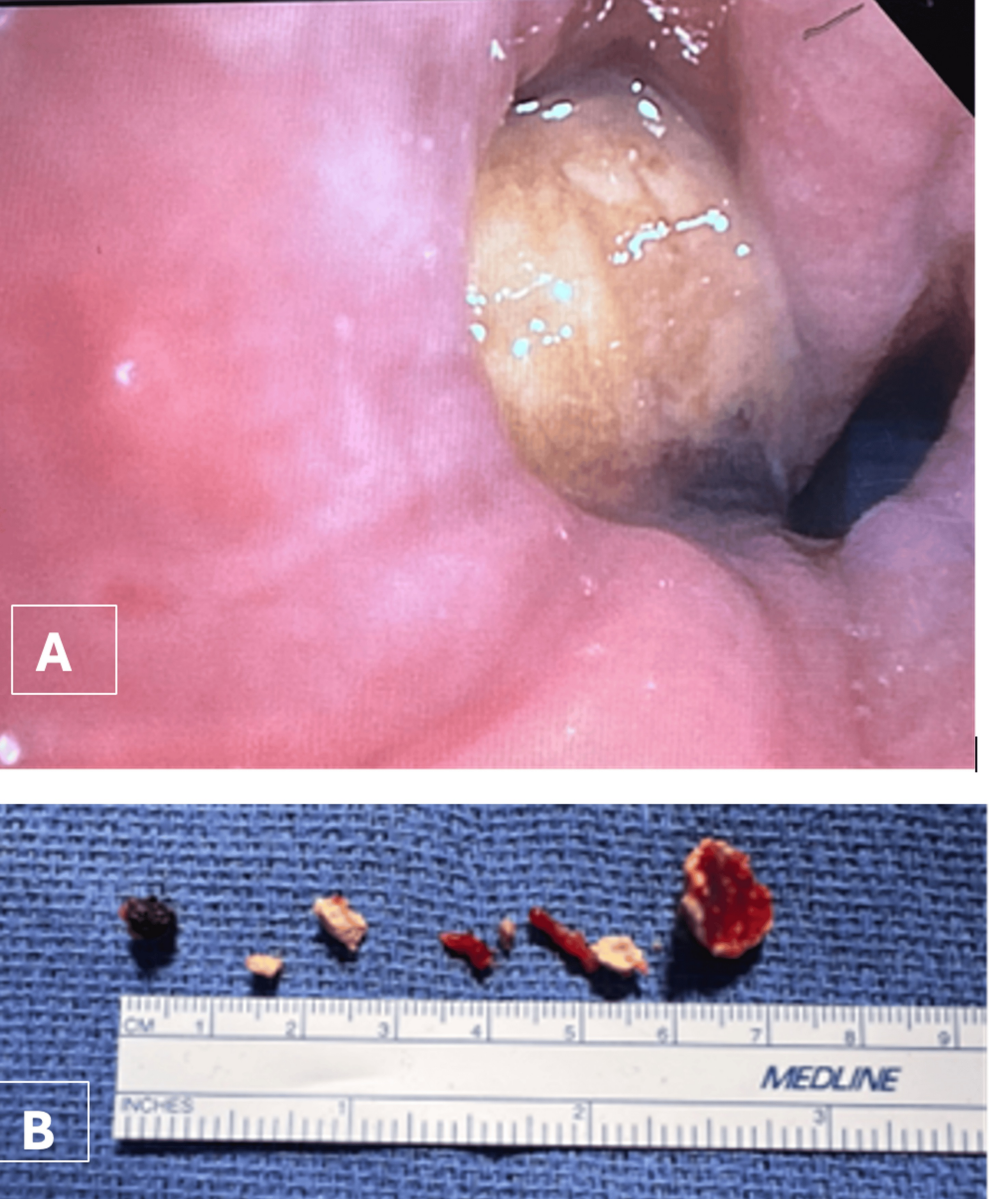
Bronchoscopic and gross findings of broncholith extraction in Case 4. Bronchoscopic view (A) demonstrating a large, adherent broncholith obstructing the left lower lobe bronchus. Gross specimen (B) showing multiple fragments of the broncholith following piecemeal extraction with forceps, displayed with a ruler for size reference.

Initial extraction attempts failed, prompting balloon dilation (CRE 8-9-10 mm) twice to 8 mm for 30 seconds. A large broncholith fragment was then removed with rat-tooth forceps, crushed, and extracted piecemeal. A second fragment was also removed, followed by repeat dilation and aspiration of a small residual broncholith (Figure 8A).

Mild hemorrhage from the superior segment was managed with epinephrine instillation (10 mL) and temporary balloon tamponade. The airway was left patent and hemostatic. CT imaging confirmed pre-procedure collapse and post-intervention patency (Figure 8B).

Case 5

**Figure 9:**
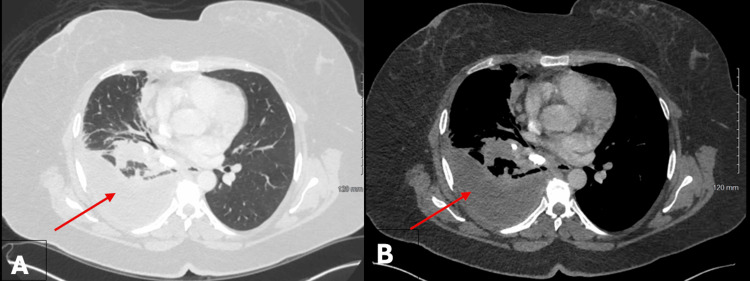
Computed tomography findings of broncholithiasis in Case 5. Axial contrast-enhanced chest CT images displayed in lung window (A) and mediastinal window (B) demonstrate a large calcified broncholith within the right lower lobe bronchus, causing complete luminal obstruction (arrows). Associated post-obstructive changes are present, with extrinsic compression of the right middle lobe bronchus.

Mrs. K, a 45-year-old female, presented with chronic cough, necrotizing pneumonia, and empyema. CT imaging revealed a 2.5 cm broncholith in the right lower lobe (RLL) causing complete obstruction and external compression of the RML (Figure 9).

**Figure 10:**
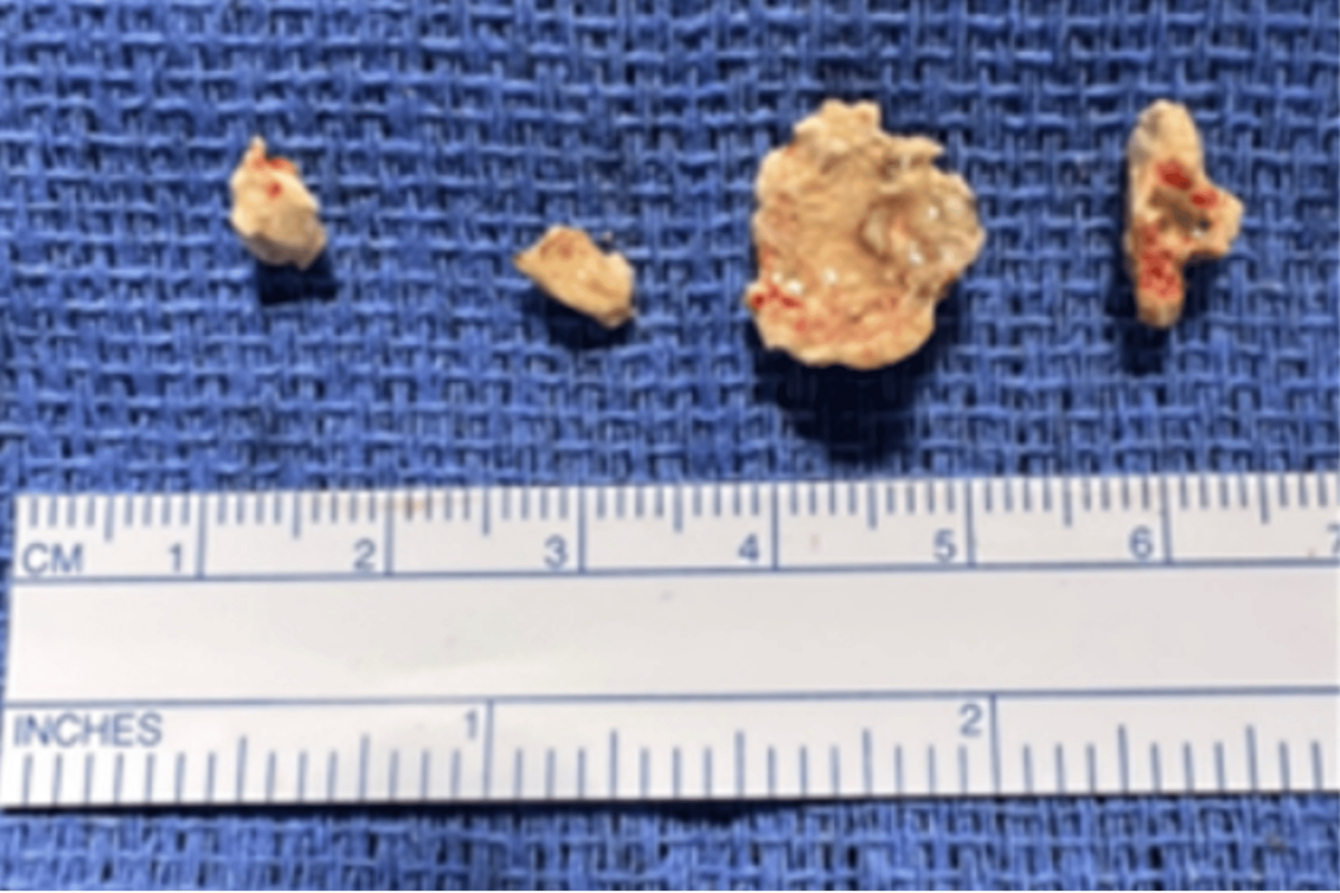
Extracted broncholith fragments in Case 5. Broncholiths extracted via bronchoscopy in pieces, demonstrating irregular shapes and variable sizes.

She underwent bronchoscopic broncholith removal (Figure 10), followed by VATS decortication and washout. Granulation tissue causing 100% RLL obstruction was debrided with pulmonary and rat-tooth forceps, cryotherapy, and APC. Serial CRE balloon dilations (8-10-12 mm) restored airway patency.

Because of its size, the broncholith was removed en bloc after extubation and subsequent reintubation. Endobronchial ultrasound (EBUS)-guided sampling of 12R lymph nodes and bronchoalveolar lavage (BAL) were performed for microbiologic and cytologic evaluation.

Post-procedure, the RLL was 50% open, and the RML remained extrinsically compressed. The patient’s respiratory symptoms improved significantly following combined bronchoscopic and surgical management.

## Discussion

Broncholithiasis is an uncommon condition most often caused by erosion of calcified mediastinal or hilar lymph nodes into the bronchial lumen following granulomatous infections, particularly histoplasmosis and tuberculosis [1,2]. Contemporary reviews emphasize its heterogeneous clinical presentation, ranging from incidental findings to complications such as recurrent infection, airway obstruction, and hemoptysis [1,3]. In older surgical series from endemic regions, cough was nearly universal, while hemoptysis and lithoptysis were reported in substantial proportions, reflecting both symptom burden and diagnostic delays characteristic of historical cohorts [4-6].

In our series, clinical presentation was heterogeneous rather than dominated exclusively by hemoptysis. Two patients presented primarily with hemoptysis, one with chronic cough accompanied by severe infectious complications, one with recurrent pneumonia and lobar collapse, and one was referred with an obstructing broncholith identified during evaluation. This distribution is more consistent with prior reports describing broncholithiasis as a variable syndrome ranging from incidental or minimally symptomatic disease to major airway obstruction, recurrent infection, and hemoptysis (Table 1) [3,7].

**Table 1:** Clinical characteristics and management of patients with broncholithiasis. Table presenting the clinical presentation and management of patients with broncholithiasis at our institution. RML: right middle lobe; EBUS: endobronchial ultrasound; BAL: bronchoalveolar lavage; APC: argon plasma coagulation; CRE: controlled radial expansion; VATS: video-assisted thoracoscopic surgery.

Case	Age/Sex	Clinical presentation	Presumed/confirmed etiology	Microbiology/pathology	Management	Follow-up/outcome	
1	70/F	Hemoptysis	Remote histoplasmosis; broncholithiasis likely secondary to prior granulomatous disease	Not reported	Rigid bronchoscopy; cryoablation; piecemeal forceps extraction; APC; epinephrine	Hemoptysis resolved; dyspnea improved at two weeks and six months	
2	60/F	Hemoptysis with recurrent infection	Underlying granulomatous etiology not definitively established; calcified mediastinal lymphadenopathy present	Cultures grew *Actinomyces* and mixed molds	Flexible bronchoscopy; CRE balloon dilation; repeat bronchoscopy; chest tube; VATS decortication; prolonged antibiotics	Gradual symptom improvement after combined bronchoscopic, surgical, and antimicrobial therapy	
3	61/M	Incidentally referred/obstructing RML broncholith	Undetermined	Not reported	Rigid bronchoscopy; Fogarty balloon dilation; fragment removal with forceps; hemostasis with thrombin and epinephrine	Clinical follow-up interval not reported	
4	82/M	Recurrent pneumonia with left lower lobe collapse	Undetermined	Not reported	Flexible bronchoscopy; balloon dilation; piecemeal extraction; epinephrine; balloon tamponade	Airway left patent and hemostatic; interval and objective follow-up not standardized in the manuscript	
5	45/F	Chronic cough with necrotizing pneumonia and empyema	Undetermined; granulomatous etiology not confirmed in text	EBUS-guided nodal sampling and BAL performed; final microbiologic/pathologic result not reported	Bronchoscopic removal; cryotherapy; APC; CRE dilation; VATS decortication and washout	Respiratory symptoms improved significantly; precise interval not reported	

Our experience supports bronchoscopy as first-line therapy in appropriately selected patients, particularly when broncholiths are intraluminal or partially eroding. Olson et al. reported uniformly successful bronchoscopic extraction for free broncholiths, while outcomes for partially eroding lesions were less favorable overall and appeared to benefit from rigid rather than flexible bronchoscopy [5]. Similarly, surgical resolution is suggested as second-line treatment, followed by anti-infectives in patients who fail removal or present recurrence, according to He et al. [7].

Compared with these historical cohorts, our series demonstrates a contemporary, escalation-based endoscopic strategy, utilizing rigid bronchoscopy when necessary, airway dilation, and adjunctive hemostatic modalities such as argon plasma coagulation and topical agents, allowing effective airway clearance without thoracotomy in all cases. This approach aligns with modern management frameworks that advocate individualized treatment based on broncholith mobility, degree of adherence, vascular proximity, and the presence of associated complications [3].

In a contemporary cohort of 63 patients, He et al. provided one of the most comprehensive modern comparisons of bronchoscopic and surgical management strategies for broncholithiasis. Bronchoscopic removal achieved high success rates for intraluminal broncholiths and acceptable outcomes for partially eroding lesions, particularly when rigid bronchoscopy and advanced endoscopic techniques were employed. Surgical intervention was primarily reserved for patients with fixed extraluminal disease, extensive airway destruction, fistulization, or severe complications such as uncontrolled hemoptysis or recurrent post-obstructive infection. Notably, surgical management was associated with greater procedural morbidity, longer hospital stays, and prolonged recovery, without a clear survival advantage [7].

Lim et al. proposed a classification of broncholiths as intraluminal, extraluminal, or mixed based on CT and bronchoscopic findings, with management outcomes varying accordingly [4]. Our procedural outcomes support this framework: lesions amenable to controlled dilation and extraction were successfully managed endoscopically, whereas the case complicated by extensive infectious sequelae required combined endoscopic and surgical management in the form of VATS decortication for empyema. Importantly, surgery was not the definitive modality for broncholith removal, supporting the concept that surgical intervention is often driven by downstream complications rather than the broncholith itself when endoscopic clearance is achievable [3].

The use of cryotherapy and argon plasma coagulation in our series is consistent with the evolving endoscopic “toolbox” described in contemporary reviews [3,6]. Beyond forceps-based extraction, newer reports describe successful removal using cryoprobes under rigid bronchoscopy and other fragmentation strategies, including laser lithotripsy in selected cases [7]. Although these techniques were not required in all patients in our series, their availability may expand the population eligible for endoscopic management and further reduce the need for surgical referral in carefully selected cases.

The differential diagnosis of an endobronchial calcified or obstructive lesion should also include carcinoid tumor and impacted foreign body, particularly when imaging or bronchoscopic appearance is atypical. Distinguishing these entities is important because management, bleeding risk, and the need for tissue diagnosis may differ substantially from true broncholithiasis.

Post-obstructive infection is an important clinical consequence of broncholithiasis and should be explicitly recognized in management planning. Chronic airway obstruction may promote retention of secretions, recurrent bacterial infection, necrotizing pneumonia, empyema, and, in selected cases, infection with organisms such as *Actinomyces* or fungal species [8]. In our series, one patient had cultures positive for *Actinomyces* and mixed molds, supporting the need to consider microbiologic evaluation when purulence, necrosis, or empyema is present. Bronchoscopic relief of obstruction is central, but antimicrobial therapy should be individualized according to culture data, radiographic complications, and the degree of pleural or parenchymal involvement.

Overall, our findings add to the growing body of evidence that, with careful patient selection, appropriate airway control, and access to advanced endoscopic adjuncts, bronchoscopic management can achieve excellent clinical outcomes while minimizing the need for thoracotomy, reserving surgery for fixed extraluminal disease, major bleeding risk, fistulization, or irreversible parenchymal destruction [1,3].

This report is limited by its retrospective single-center design and the small number of cases. In addition, follow-up was not standardized across all patients. Case 1 included clearly documented follow-up at two weeks and six months, whereas the remaining cases had outcome information recorded in less uniform terms within the medical record. Because symptom resolution, airway patency, and interval imaging were not captured at predefined time points for every patient, outcome comparisons across cases should be interpreted cautiously.

## Conclusions

Broncholithiasis, though rare, should be considered in patients presenting with hemoptysis, recurrent pneumonia, chronic cough, or other manifestations of post-obstructive airway disease, especially in regions with endemic granulomatous infections. In this case series, bronchoscopy remained the diagnostic and therapeutic cornerstone and enabled successful broncholith management in all five patients. Adjunctive surgical intervention was required only for associated pleural or infectious complications in selected cases. These findings highlight the heterogeneous clinical spectrum of broncholithiasis and the value of multidisciplinary management.
